# ILA-CSMA: Hybrid Sensing and Adaptive Fair Backoff for Large-Scale LoRa Networks

**DOI:** 10.3390/s26113593

**Published:** 2026-06-05

**Authors:** Wenjie Cheng, Haoyang Cui, Hengwen Yu

**Affiliations:** Faculty of Artificial Intelligence, Shanghai University of Electric Power, Shanghai 201306, China; 571433063@mail.shiep.edu.cn (W.C.);

**Keywords:** LoRa, LoRaWAN, wireless sensor networks, medium access control, cross-SF interference, RSSI sensing, adaptive backoff, fairness

## Abstract

Dense Long Range (LoRa) networks suffer from packet loss when many end devices contend for the same unlicensed channel. Channel activity detection (CAD) can miss weak or cross-spreading-factor (cross-SF) transmissions, while a uniform carrier sense multiple access with collision avoidance (CSMA/CA) backoff rule ignores the different time-on-air (ToA) costs of SF7–SF12 packets. To address these two coupled problems, this paper proposes an interference-limit-aware CSMA protocol (ILA-CSMA). ILA-CSMA first combines CAD with an instantaneous received signal strength indicator (RSSI) test derived from the residual interference tolerance of the selected spreading factor, and then scales the contention window according to normalized ToA. The protocol is implemented in the Framework for LoRa (FLoRa), an OMNeT++-based LoRa network simulator, and is evaluated for networks with 100–2000 nodes. Compared with Pure ALOHA, Slotted ALOHA, standard CSMA/CA, and two ablation variants, ILA-CSMA improves dense-network access by jointly reducing hidden collisions and airtime imbalance. In the 2000-node case, it increases the packet delivery ratio (PDR) by about 20 percentage points relative to standard CSMA/CA, keeps the Jain fairness index (JFI) above the 0.85 reference line, reduces the energy consumed per successful packet to 22% of the standard CSMA/CA value, and reduces conditional average packet delay from 18.5 s to 8.2 s. These results show that interference-aware sensing and ToA-aware backoff can improve large-scale LoRa access under the evaluated simulation conditions.

## 1. Introduction

LoRa is a chirp-spread-spectrum physical-layer technology, and LoRaWAN defines a network protocol stack for low-power wide-area networking in unlicensed bands [1,2]. LoRa and related low-power wide-area network technologies have been used in smart metering, environmental monitoring, industrial telemetry, and agricultural sensing, where long communication range and low device power consumption are required [3,4]. A representative dense-telemetry case is photovoltaic inverter cluster monitoring, in which many distributed terminals periodically upload operating measurements and alarm information through a limited number of gateways. This paper uses this type of application only as a motivating example; the system model and evaluation remain a general large-scale LoRa access-control setting.

Dense LoRa deployments face a channel-access problem when many end devices transmit over the same channel. Pure ALOHA and Slotted ALOHA are common reference schemes, but their collision probability increases with the offered load [5,6]. CSMA/CA can reduce part of the contention by sensing before transmission, and recent LoRaWAN studies have examined CAD-based CSMA designs for energy and fairness improvement [7]. However, CAD is not equivalent to perfect carrier sensing in LoRa networks. Weak signals, partially overlapping packets, and cross-SF interference can still produce hidden collisions [8].

A second issue is the unequal access cost caused by different SFs. Packets transmitted with larger SFs occupy the channel for a longer time and therefore have a larger collision cost. Previous analyses have shown that LoRa scalability is constrained by collisions, imperfect SF orthogonality, and airtime heterogeneity [9,10]. Fairness can be quantified by the Jain fairness index, which is widely used for shared resource allocation analysis [11]. A backoff rule that treats all SFs identically does not reflect this cost difference.

Recent reviews summarize interference, cross-layer optimization, and deployment issues in LoRaWAN and related Internet of Things systems [12,13,14,15]. Simulation tools such as FLoRa also support controlled evaluation of dense LoRa configurations and MAC-layer designs [16]. Recent optimization studies have further improved configuration, downlink operation, acknowledgment behavior, SF assignment, channel selection, and mobility support from different protocol perspectives [17,18,19,20,21,22,23,24,25]. Application studies with different payload requirements also show that LoRa access design must be interpreted together with service characteristics [26]. These studies provide useful context, but the combined problem of CAD blind spots and SF-dependent backoff cost still requires a direct MAC-layer treatment.

This paper proposes an interference-limit-aware CSMA protocol (ILA-CSMA) for large-scale heterogeneous LoRa networks. The contributions are as follows.

1. A cumulative-interference description is used to connect cross-SF interference with hidden collision risk.
2. A two-stage sensing rule combines CAD and an RSSI threshold derived from the residual interference tolerance of the selected SF.
3. A ToA-aware adaptive backoff rule scales the contention window according to normalized transmission cost.
4. The protocol is evaluated in FLoRa under 100–2000-node settings and is compared with ALOHA, Slotted ALOHA, CSMA/CA, and ablation variants.

The remainder of this paper is organized as follows. Section 2 reviews related work and clarifies the novelty of ILA-CSMA. Section 3 introduces the network model and formulates the sensing and fairness problems. Section 4 describes the proposed protocol. Section 5 presents the simulation setting and results. Section 6 concludes the paper.

## 2. Related Work and Technical Positioning

LoRaWAN scalability has been studied from several complementary directions. Early studies quantified the limits of ALOHA-like channel access and showed that collision probability grows quickly as the number of devices increases [5,6,9]. Subsequent work further showed that SFs are not perfectly orthogonal and that cross-SF interference can affect throughput and packet reception, especially in dense deployments [8,10]. Recent SF-allocation and channel-occupancy studies have continued this direction by explicitly considering intra-SF and inter-SF interference, channel occupancy, and practical dense-deployment constraints [25,27]. These results motivate MAC-layer designs that account for interference rather than treating all overlapping packets as either fully orthogonal or fully destructive.

Another line of work improves LoRaWAN configuration and fairness. ADR and SF-allocation studies tune SF, transmit power, channel, or scheduling decisions to improve capacity and fairness [16,28,29,30]. Recent studies have also considered collision-aware ADR, time-managed ADR, critical downlink service, TinyML-based channel selection, adaptive acknowledgment control, learning-assisted SF selection, and mobile ADR [17,18,19,20,21,22,23,24]. A recent systematic review also shows that AI/ML-based LoRaWAN optimization is increasingly used for energy efficiency, resource allocation, and network robustness [15]. These approaches improve different layers of LoRaWAN operation, but many of them do not explicitly combine a pre-transmission residual-interference test with an airtime-weighted contention rule at the MAC layer.

CAD-based CSMA is a natural extension of LoRa access control because it can reduce unnecessary transmissions before channel access. Recent CSMA-based LoRaWAN work has shown that carrier sensing can improve energy and fairness performance [7]. Recent CAD analysis has also revisited the LoRaWAN channel-activity-detection problem and emphasized that LoRa sensing cannot be reduced to ordinary energy detection because LoRa packets may operate at negative SNR [31]. However, CAD alone is still limited by weak signals, partial overlap, and imperfect SF orthogonality. Semtech application notes indicate that RSSI can be read during CAD and that CAD behavior depends on radio configuration, which supports hybrid sensing but does not by itself define a fairness-oriented MAC rule [32,33]. Beyond LoRa, adaptive fair MAC design has also been studied in other wireless systems; for example, velocity-adaptive access for semantic-aware vehicular networks adjusts the access window to jointly optimize fairness and Age of Information (AoI) [34]. Although the physical layer and traffic model differ from LoRaWAN, this work reinforces the broader principle that access windows should reflect heterogeneous access costs.

Table 1 summarizes the technical positioning. The main novelty of ILA-CSMA is not the isolated use of RSSI or backoff adaptation; rather, it is the coupling of (i) an SF-dependent residual-interference bound for the CAD-missed case and (ii) a ToA-normalized backoff window for airtime fairness. This combination targets the specific dense-LoRa condition in which hidden cross-SF interference and airtime imbalance occur simultaneously.

## 3. System Model and Problem Analysis

### 3.1. Network Model

**Figure 1:**
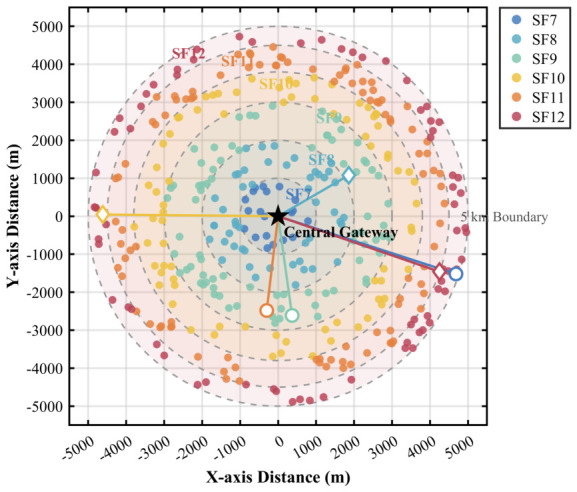
ADR-based distribution of LoRa nodes and gateway coverage boundary.

We consider a single-gateway LoRa network in which end devices are uniformly distributed in a circular area. Nodes use adaptive data rate to select SF7–SF12 according to link quality. The SF regions are not fixed hardware ranges; they are generated from the link budget used in the FLoRa simulation. The received power is computed from the transmit power, antenna gains, and the path-loss model in (1), and the lowest SF satisfying the receiver-sensitivity requirement is selected. The apparent range of each SF in Figure 1 is therefore a simulation outcome determined by propagation parameters and sensitivity thresholds, not an empirical field-measurement result. The different symbol durations and packet airtimes of SF7–SF12 create heterogeneous channel occupation. Figure 1 presents the node distribution and gateway coverage boundary obtained by drawing random node coordinates in the 5000 m circular service area and applying the same ADR-based SF-selection rule used in the simulation.

### 3.2. Propagation and Cross-SF Interference

The large-scale path loss is modeled in the logarithmic form

(1) $$PL\left(d\right)=PL\left(d_{0}\right)+10n\log_{10}\;\left(\frac{d}{d_{0}}\right)+X_{\sigma }.$$

In (1), PL(d) is the path loss at distance *d*, $PL(d_{0})$ is the path loss at the reference distance $d_{0}$, *n* is the path loss exponent, and $X_{\sigma }$ is the shadowing term. The logarithmic distance dependence is used because average received power in large-scale wireless propagation decays approximately as a power law with distance; after conversion to decibels, this power-law relationship becomes linear in $\log_{10}\left(d/d_{0}\right)$. The random term $X_{\sigma }$ represents slow shadowing caused by buildings, terrain, or vegetation. This model is widely used for system-level wireless simulation because it captures distance-dependent attenuation without requiring site-specific ray tracing.

Although LoRa SFs can be separated at the receiver, practical orthogonality is not ideal. When several transmissions overlap in time, the aggregate interference in the gateway reception model is first accumulated in linear power units as

(2) $$I_{\mathrm{agg}}^{\mathrm{lin}}=\sum_{j\in S}P_{r,j}^{\mathrm{lin}}.$$

In (2), $I_{\mathrm{agg}}^{\mathrm{lin}}$ is the aggregate received interference power, S is the set of concurrent interferers, and $P_{r,j}^{\mathrm{lin}}$ is the received power contributed by interferer *j*. For comparison with RSSI and receiver margins, this value is converted to dBm as $I_{\mathrm{agg}}=10\log_{10}\left(I_{\mathrm{agg}}^{\mathrm{lin}}/1\;\mathrm{mW}\right)$.

**Figure 2:**
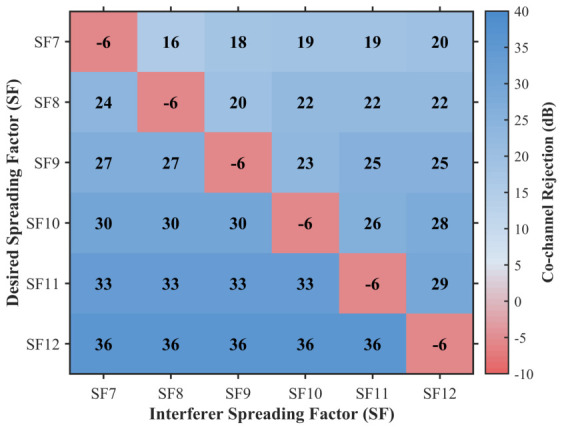
Co-channel rejection matrix used to characterize interference suppression among spreading factors.

If $I_{\mathrm{agg}}$ is not detected by CAD but is still large enough to reduce the demodulation margin, a hidden collision may occur. Coexistence with neighboring sub-GHz systems and new PHY options such as LR-FHSS also changes the interference budget that must be considered in dense deployments [36,37]. Figure 2 gives the co-channel rejection relationship used to represent different cross-SF interference tolerances. The matrix was generated from cross-SF rejection values reported in LoRa interference studies and implemented as a lookup table in the simulator. Each row denotes the target packet SF, each column denotes the interfering packet SF, and each cell gives the desired-to-interferer power separation used for reception judgment. The matrix shows that tolerated interference depends on the SF combination and is not symmetric across all SF pairs.

### 3.3. Transmission Cost and Fairness

The ToA of a LoRa packet increases with SF. A large-SF packet therefore consumes more channel time for one transmission attempt and also loses more channel time when a collision occurs. To represent this difference, the normalized transmission cost of node *i* is defined as

(3) $$w_{i}=\frac{{\mathrm{ToA}}_{i}}{\max_{k}{\mathrm{ToA}}_{k}}.$$

In (3), $w_{i}$ is the normalized transmission cost of node *i*, ${\mathrm{ToA}}_{i}$ is the airtime of node *i*, and $\max_{k}{\mathrm{ToA}}_{k}$ is the maximum airtime among all nodes indexed by *k*.

**Figure 3:**
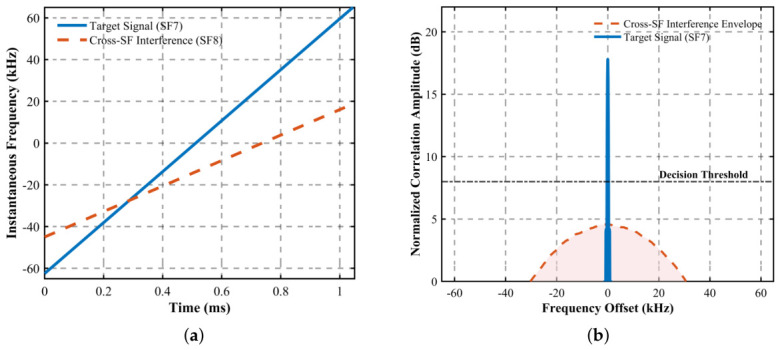
Signal-domain view of cross-SF interference and de-spreading behavior. (**a**) Instantaneous frequency evolution of the target and interfering signals. (**b**) Normalized amplitude of the de-spread signal in the correlation domain.

When all nodes use the same backoff rule, nodes with short airtime can re-enter contention more frequently than nodes with long airtime. This effect can reduce access fairness under high load. Prior work on SF and power allocation, scheduling, and fairness-aware access control also indicates that LoRa performance should be evaluated with airtime imbalance and imperfect orthogonality considered together [28,35]. Figure 3 provides a signal-domain interpretation of cross-SF interference. Figure 3a was generated from idealized LoRa up-chirp trajectories with different SF-related symbol durations, while Figure 3b was generated by de-spreading the superposed target and interfering components and normalizing the resulting correlation amplitude. The illustration highlights why overlapping chirps with different SFs can still leave nonzero residual components after de-spreading and why CAD alone may fail to prevent some hidden collisions.

## 4. ILA-CSMA Protocol Design

### 4.1. Overall Architecture

**Figure 4:**
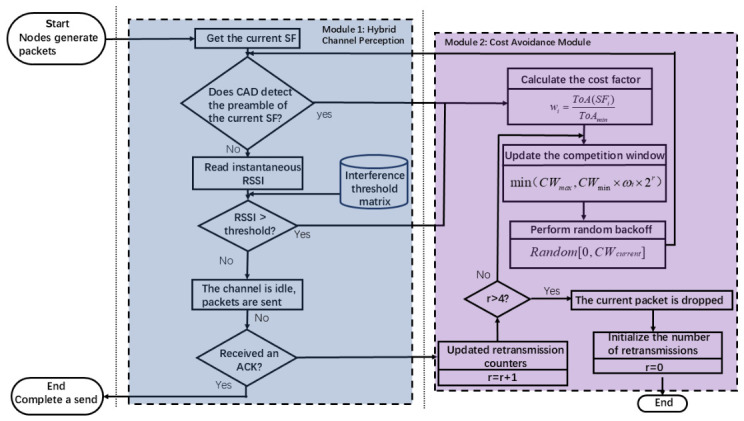
Overall execution workflow of the proposed ILA-CSMA protocol.

ILA-CSMA contains two coordinated parts. The first performs channel assessment using CAD and RSSI; the second computes a backoff window from the congestion stage and normalized ToA. This structure maps the two observations in Section 3 into a reproducible protocol rule: the sensing step checks whether residual interference exceeds the SF-dependent tolerance, and the backoff step adjusts retransmission timing according to airtime cost. Figure 4 presents the execution flow, including packet generation, channel assessment, RSSI threshold checking, transmission, acknowledgment processing, and backoff updating. The workflow emphasizes that ILA-CSMA does not rely on a single sensing result; the transmission decision combines CAD, interference-threshold comparison, and the subsequent backoff adjustment.

### 4.2. Two-Stage Hybrid Sensing

**Figure 5:**
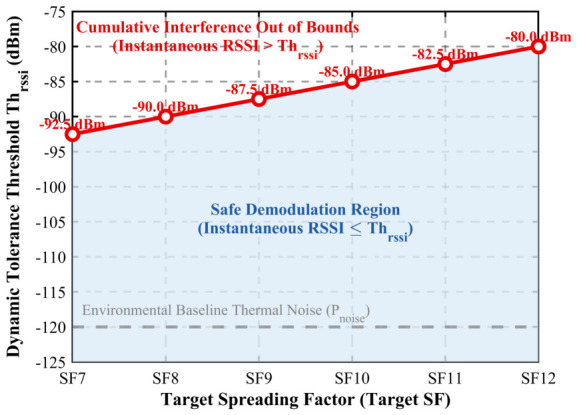
Dynamic tolerance thresholds for cumulative cross-SF interference under different spreading factors.

The first sensing stage performs standard CAD. If CAD reports a busy channel, the node defers transmission. If CAD reports an idle channel, the second stage reads instantaneous node-side RSSI and compares the measured channel-activity estimate with a dynamic threshold. This design follows Semtech application guidance showing that RSSI can be read during CAD and used with CAD to support channel assessment [32,33]. Figure 5 shows how the threshold varies with SF. The curves correspond to the SF-dependent interference tolerances used in the RSSI-based sensing rule for SF7–SF12. The figure indicates that the interference bound applied before transmission is determined jointly by the selected SF and the corresponding reception margin, rather than by a single fixed RSSI threshold for all packets.

The residual interference threshold used by the second sensing stage is defined as

(4) $$I_{i}^{\mathrm{th}}=P_{\mathrm{sig},i}-\Gamma_{\mathrm{SF}(i)}.$$

In (4), $I_{i}^{\mathrm{th}}$ is the maximum residual interference allowed for node *i*, $P_{\mathrm{sig},i}$ is its expected gateway-side received signal power, and $\Gamma_{\mathrm{SF}(i)}$ is the SF-dependent interference margin. The expression follows from a desired-signal-to-interference protection condition. For a packet transmitted by node *i*, successful demodulation requires

(5) $$P_{\mathrm{sig},i}-I_{\mathrm{agg}}\geq \Gamma_{\mathrm{SF}(i)},$$

where all power quantities are in dB or dBm units, and $\Gamma_{\mathrm{SF}(i)}$ denotes the protection margin associated with the selected SF and the cross-SF rejection table. Rearranging (5) gives $I_{\mathrm{agg}}\leq P_{\mathrm{sig},i}-\Gamma_{\mathrm{SF}(i)}$, which yields (4). Thus, the threshold is not an arbitrary fitted parameter. $P_{\mathrm{sig},i}$ is obtained from the simulation link budget, while $\Gamma_{\mathrm{SF}(i)}$ is derived from receiver sensitivity and cross-SF rejection values used in LoRa interference modeling [8,10]. In practical hardware, the same rule can be calibrated by replacing the simulation lookup table with measured rejection margins for a given radio and bandwidth.

The node transmits only when the measured aggregate interference estimate satisfies

(6) $$I_{\mathrm{agg}}<I_{i}^{\mathrm{th}}.$$

In (6), $I_{\mathrm{agg}}$ is the aggregate interference estimate defined in (2), and $I_{i}^{\mathrm{th}}$ is the threshold defined in (4).

The rule does not require the node to identify every interferer. It only estimates whether residual channel activity is below the tolerance assigned to the current SF. In the simulation, this estimate is mapped to the same dBm scale as the gateway-side interference model; in a distributed implementation, it would be obtained from the local RSSI sample and would remain a proxy rather than a perfect measurement of gateway interference. This assumption follows the reported behavior of imperfect SF orthogonality and cross-SF overlap, especially when several transmissions overlap [10]. Figure 5 illustrates the SF-dependent threshold used by the second sensing stage. The plotted values show that the permissible residual interference changes with the reception margin assigned to each SF; consequently, a channel condition judged acceptable for one SF may still be unsuitable for another SF. The threshold values are validated here by consistency with published cross-SF rejection behavior and by the ablation comparison in Section 5.2; hardware calibration is left for future experimental work.

### 4.3. Protocol Overhead and Implementation Feasibility

ILA-CSMA introduces one additional RSSI read at the transmitting node and one contention-window calculation after a successful CAD-idle result. The sensing latency overhead is therefore bounded by one CAD operation plus one RSSI sampling step. With the 125 kHz bandwidth used in the evaluation, a two-symbol CAD operation lasts about 2.05 ms at SF7 and about 65.54 ms at SF12 before implementation-specific radio turnaround; the RSSI register read and the fixed-point contention-window calculation are sub-millisecond operations relative to packet airtime. The RSSI value is already available in common LoRa transceivers during CAD operation, so the proposed hybrid sensing does not require an additional receiver chain [32,33]. The computation overhead is also small: (4) requires one subtraction in dB units, and (7) requires one multiplication, one power-of-two shift or lookup, one ceiling operation, one maximum operation, and one minimum operation. These operations can be implemented with fixed-point arithmetic or precomputed tables indexed by SF and retransmission stage.

The energy overhead comes mainly from keeping the radio in receive or CAD mode for the extra RSSI check. This cost is traded against the energy saved by avoiding transmissions that are likely to fail under excessive residual interference. Under light load, the additional check can slightly increase access latency and sensing energy. Under dense load, the reduction in failed transmissions dominates this overhead in the simulation results. The implementation remains feasible for resource-constrained LoRa devices because it uses existing CAD/RSSI radio functions and simple MAC-layer arithmetic rather than continuous spectrum sensing or centralized scheduling.

### 4.4. Cost-Aware Adaptive Backoff

When the channel is judged busy or when an expected acknowledgment is not received, the node enters random backoff before the next transmission attempt. Standard binary exponential backoff applies the same window rule to all devices. ILA-CSMA scales the contention window by the normalized transmission cost in (3), so that long-airtime packets use a larger contention range under congestion. The contention window for node *i* at retransmission stage *r* is

(7) $$CW_{i}^{\left(r\right)}=\min\;\left(CW_{\max},\max\;\left(CW_{\min},\left\lceil w_{i}2^{r}CW_{\min}\right\rceil \right)\right).$$

In (7), $CW_{i}^{\left(r\right)}$ is the contention window of node *i* at stage *r*, $CW_{\min}$ is the minimum contention window, $CW_{\max}$ is the maximum contention window, $w_{i}$ is the normalized transmission cost from (3), and ⌈·⌉ is the ceiling operator. The inner maximum keeps the actual contention window no smaller than $CW_{\min}$, while the ToA factor increases the window of long-airtime packets as the retransmission stage grows.

The actual backoff duration is then selected as

(8) $$T_{\mathrm{bo}}=kT_{\mathrm{slot}},\;k∼U\left\{0,\ldots ,CW_{i}^{\left(r\right)}-1\right\}.$$

In (8), $T_{\mathrm{bo}}$ is the backoff duration, $T_{\mathrm{slot}}$ is the basic backoff slot length, *k* is the randomly selected slot count, and $U\{0,\ldots ,CW_{i}^{\left(r\right)}-1\}$ denotes a discrete uniform distribution over the stated integer set.

**Figure 6:**
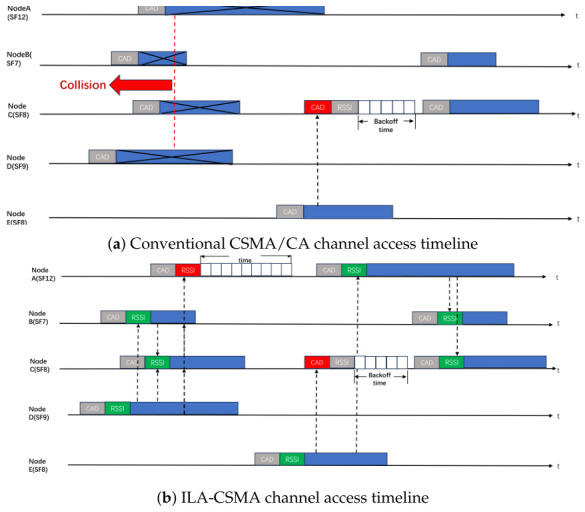
Channel access timelines under conventional CSMA/CA and the proposed ILA-CSMA.

After a successful transmission, *r* is reset to zero. After a failed transmission or repeated deferral, *r* increases until the retry limit or $CW_{\max}$ is reached. Studies on SF allocation and fair ADR also treat airtime imbalance as a fairness factor in LoRaWAN [29,30]. Figure 6 compares the access timelines of conventional CSMA/CA and ILA-CSMA. In the ILA-CSMA timeline, the additional RSSI check and the cost-aware backoff change the order and duration of sensing, waiting, and retransmission steps. The comparison shows that the proposed method inserts one more interference-screening step before transmission and allocates contention time in a way that reflects transmission cost, so repeated access attempts are separated more deliberately than in the conventional timeline.

## 5. Performance Evaluation

### 5.1. Simulation Setup

The protocol is evaluated in OMNeT++ using the FLoRa framework [16]. OMNeT++/FLoRa was selected because it provides an event-driven LoRaWAN simulation stack, configurable radio and energy models, ADR support, and packet-level statistics that can be extended at the MAC layer. Compared with a purely analytical model, it captures asynchronous packet overlap and retransmission behavior; compared with a hardware-only testbed, it permits repeatable 100–2000-node experiments with identical traffic and topology seeds. This choice is therefore appropriate for isolating the MAC-layer effects of the proposed sensing and backoff rules, although hardware validation remains necessary before deployment.

The simulation uses one gateway and 100–2000 nodes randomly distributed within a radius of 5000 m. This setting abstracts dense telemetry applications, such as photovoltaic inverter cluster monitoring, where many field terminals periodically report measurements through shared LoRa gateways. Nodes use a 470 MHz carrier, coding rate 4/8, and SF7–SF12. Each simulation lasts 10,000 s, and each configuration is repeated 10 times. Pure ALOHA, Slotted ALOHA, and standard CSMA/CA are used as baselines because they separate the effects of unslotted random access, slotted random access, and carrier sensing in LoRa networks [5,6,7]. Table 2 lists the common simulation parameters before the results are discussed.

All compared protocols use the same uplink traffic process, packet format, retry policy, ADR logic, and radio parameters. Only the channel-access decision and backoff calculation differ among the CSMA variants. The metrics are packet delivery ratio (PDR), effective channel utilization, Jain fairness index (JFI), average energy consumption per successfully delivered packet, conditional average delay of successfully delivered packets, and received signal-to-noise ratio (SNR). The SNR is recorded at the gateway for successfully decoded uplink packets and is used as a physical-layer diagnostic metric; it is not directly optimized by the MAC protocol but helps verify that performance gains are caused by collision reduction rather than by changing the link budget.

For reproducibility, PDR is computed as $N_{\mathrm{succ}}/N_{\mathrm{gen}}$, where $N_{\mathrm{gen}}$ and $N_{\mathrm{succ}}$ are the generated and successfully delivered uplink packets, respectively. Effective channel utilization is defined as

(9) $$U_{\mathrm{eff}}=\frac{\sum_{p\in P_{\mathrm{succ}}}{\mathrm{ToA}}_{p}}{T_{\mathrm{sim}}},$$

where $P_{\mathrm{succ}}$ is the set of successfully delivered packets and $T_{\mathrm{sim}}$ is the simulation time; failed airtime is therefore not counted as useful utilization. The delay metric is conditional on successful delivery and is not used alone to rank protocols with very different PDR values. The energy metric is $E_{\mathrm{succ}}=E_{\mathrm{total}}/N_{\mathrm{succ}}$. Jain fairness is computed as

(10) $$J\left(x\right)=\frac{{\left(\sum_{q=1}^{m}x_{q}\right)}^{2}}{m\sum_{q=1}^{m}x_{q}^{2}}.$$

For the global JFI in Figure 9, $x_{q}$ denotes node-level success probability. For the cross-SF JFI in Figure 15, $x_{q}$ denotes the PDR of one SF group. The useful-airtime contribution in Figure 13 is

(11) $$C_{s}=\frac{\sum_{p\in P_{\mathrm{succ},s}}{\mathrm{ToA}}_{p}}{\sum_{r=7}^{12}\sum_{p\in P_{\mathrm{gen},r}}{\mathrm{ToA}}_{p}},$$

where s∈{7,…,12} is the SF group, and *r* indexes all SF groups. This metric measures the fraction of total offered airtime that becomes useful delivered airtime in each SF group. Unlike per-SF PDR, it keeps the ToA weighting in the numerator and uses an all-SF offered-airtime denominator, so it shows how each SF contributes to useful channel occupation.

### 5.2. Results and Discussion

The comparison includes two ablation variants. CSMA-AB uses only the cost-aware adaptive backoff, and CSMA-HS uses only the two-stage hybrid sensing. These variants separate the contribution of sensing from that of backoff control.

**Figure 7:**
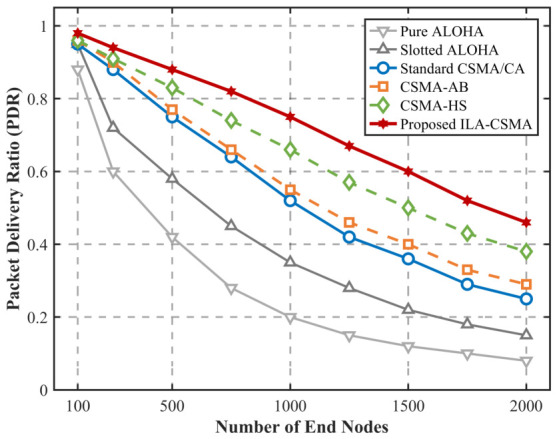
Packet delivery ratio under different network sizes for the compared protocols.

The PDR curves in Figure 7 provide the first comparison. Under low node density, all carrier-sensing schemes show similar PDR because channel contention is limited. As the node count increases, hidden collisions and repeated retransmissions become more frequent. In the 2000-node case, ILA-CSMA improves PDR by about 20 percentage points relative to standard CSMA/CA. CSMA-HS accounts for most of this improvement because it screens transmissions with excessive residual interference, whereas CSMA-AB alone cannot address CAD misses.

The received SNR distributions were also checked for the compared CSMA variants. Because all protocols use the same topology, transmit power, propagation model, and ADR logic, their median SNR values remain close for the same node-density setting. The main difference is that ILA-CSMA removes a larger fraction of attempts made under high residual-interference conditions before transmission. Therefore, the PDR improvement should be interpreted as a MAC-layer collision-avoidance gain under comparable link-budget conditions, not as an SNR gain caused by different physical-layer parameters.

**Figure 8:**
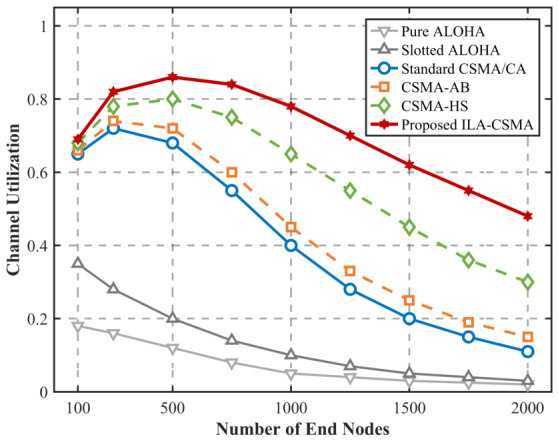
Effective channel utilization under different network sizes for the compared protocols.

Effective channel utilization, plotted in Figure 8, follows (9) and counts only the airtime that results in delivered packets. In dense settings, standard CSMA/CA and CSMA-AB spend more airtime on packets that later fail because of hidden interference. CSMA-HS reduces this loss by adding the RSSI threshold, and ILA-CSMA further separates retransmissions in time through ToA-aware backoff. In the 2000-node case, ILA-CSMA reaches an effective channel utilization of 48%.

**Figure 9:**
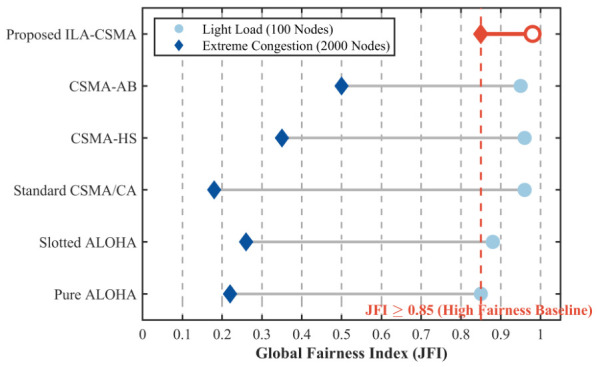
Global node-level Jain fairness index at 100 and 2000 nodes for the compared protocols.

The fairness comparison in Figure 9 shows a lower JFI for standard CSMA/CA at high node density because high-SF nodes experience longer packet airtime and higher collision cost. CSMA-HS improves packet delivery but does not directly adjust contention among nodes with different airtime. CSMA-AB adjusts contention but cannot address interference missed by CAD. ILA-CSMA combines both effects and keeps JFI above the 0.85 reference line in the tested dense settings, while the plotted marker is capped at the right edge of the index range. Table 3 further interprets this result across SFs. Lower SF nodes mainly benefit from reduced contention with long-airtime retransmissions, whereas higher SF nodes benefit from fewer repeated collisions and from contention windows that explicitly account for their larger ToA. The resulting success probabilities are less skewed across SF7–SF12 than under standard CSMA/CA.

**Figure 10:**
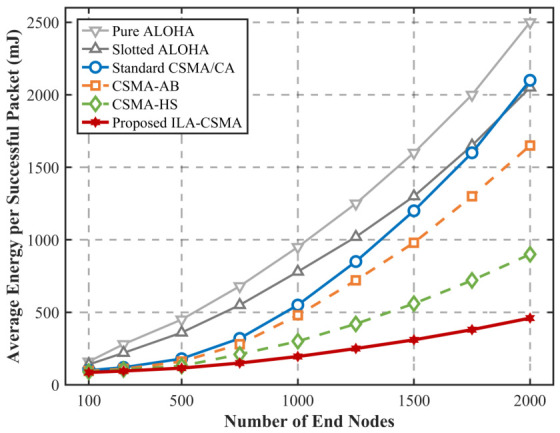
Average energy consumption per successfully delivered packet for the compared protocols.

Energy consumption per delivered packet follows the collision and retransmission trends in Figure 10. Failed transmissions consume energy without contributing to delivered packets, so the energy cost rises when collisions and retransmissions increase. CSMA-HS reduces invalid transmissions, CSMA-AB spreads retransmission attempts, and ILA-CSMA combines these two effects. In the 2000-node case, the energy consumed per successful packet is 22% of the standard CSMA/CA value.

**Figure 11:**
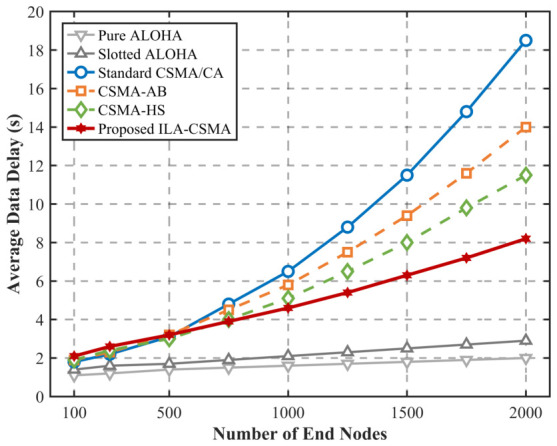
Average delay of successfully delivered packets under different network sizes for the compared protocols.

Delay is affected by both sensing and backoff, as shown in Figure 11. The reported value is the conditional average over successfully delivered packets, so it must be interpreted together with PDR rather than as a stand-alone latency guarantee. Under light load, the additional sensing step and the larger windows can increase access delay. Under high load, collision reduction can offset this increase by reducing repeated retransmissions. In the 2000-node case, the conditional average delay of ILA-CSMA is 8.2 s, while the corresponding value of standard CSMA/CA is 18.5 s. The lower delay of Pure ALOHA and Slotted ALOHA in the same figure is not a throughput advantage, because those protocols deliver far fewer packets under heavy load.

#### SF-Level, SNR, and Ablation Diagnostics

The aggregate trends are then decomposed through SF-level delivery, useful airtime contribution, node-level success probability, cross-SF fairness, received SNR, and ablation diagnostics. These diagnostics address airtime heterogeneity, physical-layer comparability, and component-level contribution in the evaluation.

**Figure 12:**
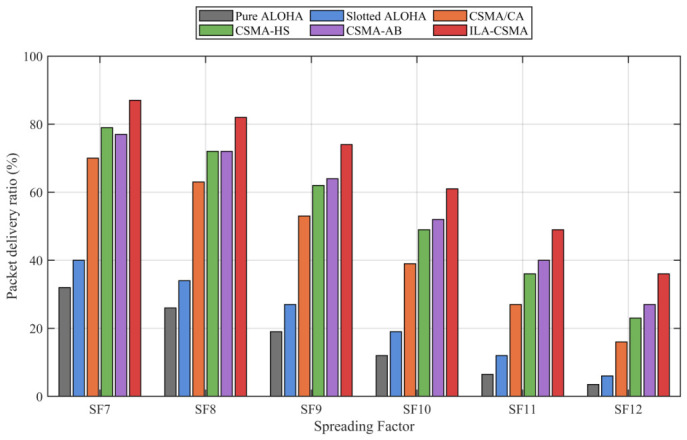
Per-SF packet delivery ratio under the 2000-node dense-deployment scenario.

Figure 12 decomposes dense-load PDR by SF. Under standard CSMA/CA, the delivery ratio declines from the low-SF groups to the high-SF groups because SF11 and SF12 packets occupy the channel for a longer time and are exposed to a longer vulnerable period. CSMA-HS improves the PDR of most SF groups by suppressing transmissions under excessive residual interference, whereas CSMA-AB mainly improves medium- and high-SF access by spacing retransmission attempts according to airtime cost. ILA-CSMA provides the most stable per-SF delivery profile: the low-SF delivery ratios remain high, while the SF10–SF12 groups are substantially improved compared with standard CSMA/CA. Thus, the aggregate PDR gain in Figure 7 is accompanied by a reduction in SF-dependent delivery imbalance.

**Figure 13:**
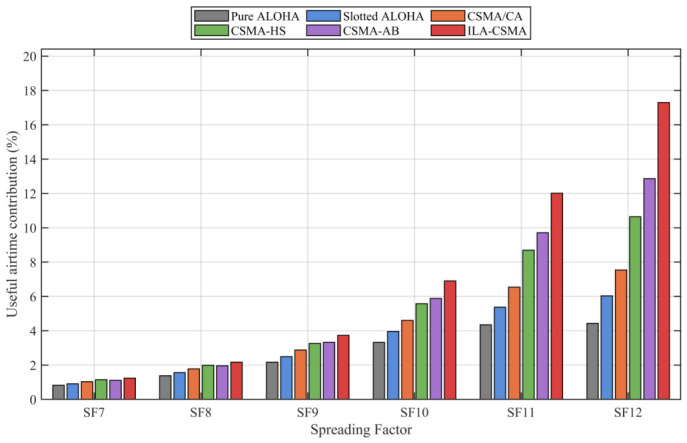
Per-SF useful airtime contribution under the 2000-node dense-deployment scenario.

The useful-airtime contribution in Figure 13 provides an airtime-aware view of the same fairness problem and is computed using (11). Because different SFs have different ToA values, packet-level PDR alone does not show how much useful channel occupation each SF contributes. Standard CSMA/CA allocates a smaller useful-airtime share to SF11 and SF12 because long-airtime packets suffer more repeated collisions under dense contention. CSMA-HS improves this share by screening residual interference, while CSMA-AB improves it by spacing retransmissions according to airtime cost. ILA-CSMA gives the most balanced useful-airtime profile among the compared protocols, indicating that hybrid sensing and ToA-aware backoff improve airtime-level balance rather than only increasing packet-level delivery.

**Figure 14:**
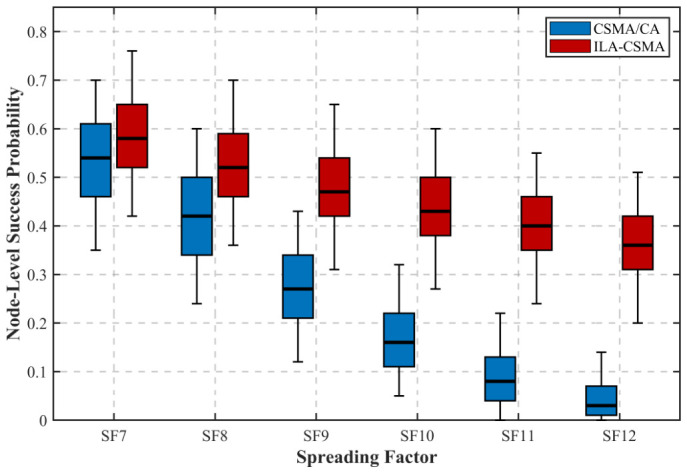
Node-level success-probability distribution for SF7–SF12 nodes under the 2000-node scenario.

Figure 14 gives the distribution of node-level success probability within each SF group. Compared with standard CSMA/CA, ILA-CSMA raises the median success probability for all SF groups and produces a particularly large improvement for SF10–SF12. The high-SF boxes shift upward and become less concentrated near low success probability values, indicating improvement in both mean delivery performance and the reliability experienced by individual long-airtime nodes. This node-level evidence complements the aggregate JFI in Figure 9.

**Figure 15:**
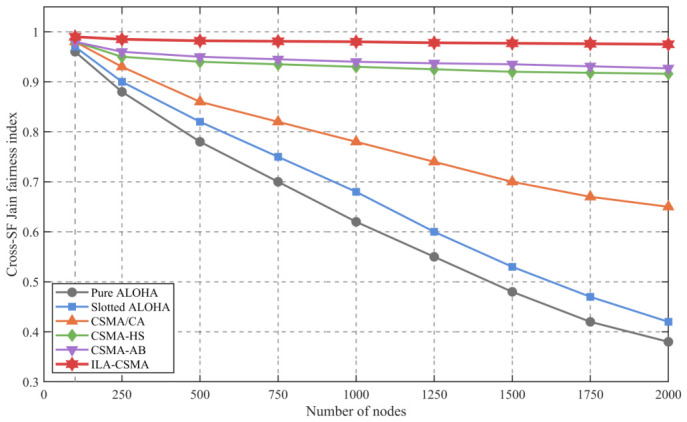
Cross-SF Jain fairness index based on SF-group PDR under increasing network density.

Figure 15 reports the Jain fairness index computed across SF groups using (10) with each SF-group PDR as one element of x. All protocols experience some fairness degradation as the number of nodes increases, but the degradation rate differs substantially. The ALOHA-based baselines show lower cross-SF fairness under heavy load because long-airtime high-SF packets lose a larger fraction of delivery opportunities. Among the CSMA-based protocols, standard CSMA/CA decreases most rapidly because a uniform contention rule does not compensate for the larger collision exposure of high-SF packets. CSMA-HS and CSMA-AB each improve fairness through one mechanism only: interference screening or ToA-aware spacing. ILA-CSMA maintains the highest cross-SF JFI throughout the tested density range, demonstrating that hybrid sensing and adaptive fair backoff jointly reduce SF-dependent access imbalance.

**Figure 16:**
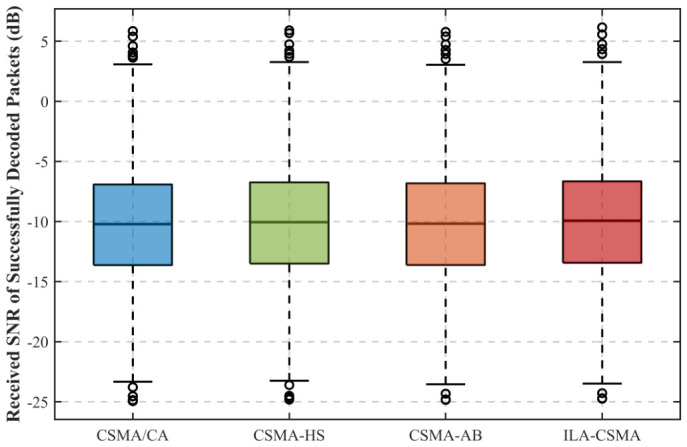
Received SNR distribution of successfully decoded packets under the 2000-node scenario.

Figure 16 reports the gateway-side SNR distribution for successfully decoded packets. The four CSMA variants have similar median SNR values and comparable interquartile ranges because the same topology, link budget, propagation model, and ADR rule are used for all protocols. The small differences among the lower tails are consistent with random packet-selection effects after residual-interference screening, but the figure does not indicate any artificial physical-layer advantage. The distribution instead supports the interpretation that the improvements in PDR, energy consumption, and delay mainly come from MAC-layer collision avoidance and retransmission reduction.

**Figure 17:**
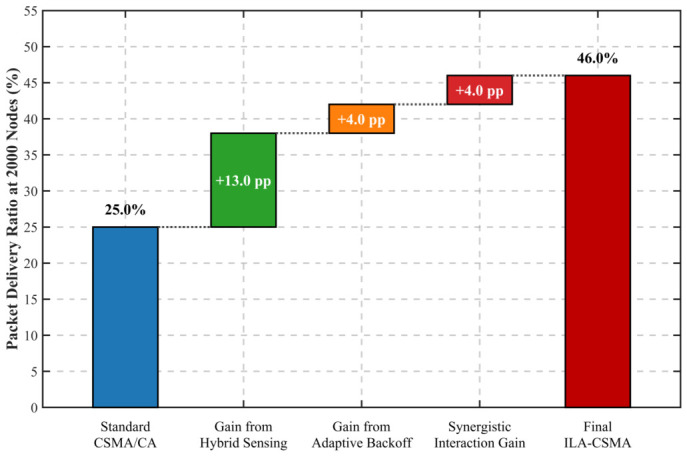
Ablation-based performance-gain breakdown for the 2000-node scenario.

The ablation breakdown in Figure 17 summarizes component contributions under the most congested scenario. Starting from standard CSMA/CA, the hybrid-sensing component contributes the largest individual improvement because it suppresses transmissions that would otherwise be attempted after CAD misses. The adaptive-backoff component provides an additional gain by reducing repeated contention among long-airtime packets. The final ILA-CSMA result also contains a synergistic gain, indicating that residual-interference screening and ToA-aware retransmission spacing reinforce each other under dense load. Taken together, the ablation results support the design premise that ILA-CSMA is a coupled MAC-layer treatment of hidden interference and airtime fairness, rather than a simple aggregation of two independent heuristics.

Table 4 summarizes representative high-density results. In addition to the aggregate metrics, Figure 12, Figure 13, Figure 14 and Figure 15 show that the proposed method improves SF-level delivery, useful airtime contribution, and cross-SF fairness, while Figure 16 confirms that these gains are obtained under comparable received-SNR conditions.

These results are limited to the single-gateway simulation setting and the traffic assumptions in Table 2. Multi-gateway reception, synchronization error in slotted access, and detailed downlink scheduling are not modeled. The reported gains should therefore be interpreted as simulation evidence for the tested dense-access conditions rather than as deployment-independent guarantees.

## 6. Conclusions

This paper presented ILA-CSMA, a dense-LoRa MAC protocol that jointly addresses CAD blind spots and SF-dependent airtime imbalance. The protocol first applies a two-stage sensing rule that combines CAD with an RSSI-based residual-interference threshold derived from the selected SF and the cross-SF rejection margin. It then applies a ToA-aware adaptive backoff rule so that retransmission timing reflects the channel-occupation cost of each packet. Compared with conventional CAD-based CSMA, the proposed method treats hidden residual interference and airtime fairness as coupled MAC-layer decisions rather than as separate enhancements.

The FLoRa evaluation shows that this coupling improves dense-network performance under the tested assumptions. In the 2000-node setting, ILA-CSMA increases PDR by about 20 percentage points relative to standard CSMA/CA, maintains JFI above the 0.85 reference line, reduces the energy consumed per successful packet to 22% of the standard CSMA/CA value, and lowers the conditional average delay from 18.5 s to 8.2 s. The ablation variants further indicate that hybrid sensing mainly reduces hidden collision risk, while ToA-aware backoff improves fairness and retransmission spacing. The SNR check confirms that these gains are obtained under comparable link-budget conditions rather than by changing physical-layer parameters.

Several limitations remain. The evaluation is simulation-based, uses a single-gateway topology, and assumes the radio and traffic parameters listed in Table 2. Hardware measurements are still needed to calibrate the RSSI threshold, quantify CAD/RSSI latency on specific transceivers, and verify performance under real interference and clock uncertainty. Future work will evaluate ILA-CSMA in multi-gateway deployments, dynamic traffic models, LR-FHSS coexistence, mobile ADR scenarios, and hardware-in-the-loop or testbed experiments.

## Figures and Tables

**Table 1:** Comparison with representative related access-control approaches.

Approach	Main Mechanism	Limitation for Dense LoRa	Difference of ILA-CSMA
ALOHA/Slotted ALOHA [5,6]	Random or slotted random access	High collision probability under dense load	Adds sensing and adaptive contention before transmission
CAD-based CSMA/CAD analysis [7,31]	CAD before transmission or CAD algorithm modeling	CAD may miss weak or cross-SF interference	Adds RSSI-based residual- interference checking
ADR/SF/channel allocation [16,23,25,27,29]	Link-adaptive SF, channel, or power selection	Does not directly control each contention attempt	Uses the selected SF to set sensing and backoff rules
Fair scheduling/fair ADR [30,35]	Fair resource or data- rate allocation	May require scheduling assumptions or does not handle CAD misses	Uses distributed ToA-aware backoff with hybrid sensing
Velocity-adaptive fair MAC [34]	Access window adjusted by mobility and AoI cost	Designed for semantic V2X, not LoRa PHY/CAD	Adapts the same cost-aware idea to LoRa airtime and cross-SF interference
Proposed ILA-CSMA	CAD +RSSI sensing and ToA-aware backoff	Evaluated so far by simulation only	Jointly treats hidden interference and SF airtime imbalance

**Table 2:** Core simulation parameters.

Parameter	Value
Topology	Single gateway; uniformly distributed end devices
Coverage radius	5000 m
Number of nodes	100, 250, 500, 750, 1000, 1250, 1500, 1750, and 2000
Carrier frequency	470 MHz
Bandwidth	125 kHz
Transmit power and antenna gains	14 dBm; 0 dBi transmitter/receiver antenna gains
Path-loss model	Log-distance model; $d_{0}=1$ m, n=2.7, $X_{\sigma }∼N\left(0,4^{2}\right)$ dB
Noise floor	−117 dBm, including receiver noise figure
Receiver sensitivity	−123, −126, −129, −132, −134.5, and −137 dBm for SF7–SF12
Bandwidth/coding/preamble	125 kHz, coding rate 4/8, 8-symbol preamble
Spreading factor assignment	SF7–SF12 selected by the common ADR/link-budget rule
Payload size	20 bytes
Traffic source	Periodic uplink with a 300 s reporting interval and random initial offset; identical offered load for all protocols
Acknowledgment and retry policy	ACK enabled; maximum 3 retransmission attempts
CAD and RSSI sensing	CAD duration of 2 LoRa symbols; one RSSI read after a CAD-idle result for ILA-CSMA/CSMA-HS
Backoff parameters	$T_{\mathrm{slot}}=20$ ms, $CW_{\min}=8$, and $CW_{\max}=1024$ slots for CSMA variants
Energy model	3.3 V supply; TX/RX/CAD/sleep currents of 28 mA, 10.8 mA, 10.8 mA, and 1 μA
Simulation time	10,000 s
Independent runs	10 random seeds per configuration
Compared protocols	Pure ALOHA, Slotted ALOHA, CSMA/CA, CSMA-HS, CSMA-AB, and ILA-CSMA

**Table 3:** Per-SF fairness interpretation under dense contention.

SF Group	Main Imbalance UnderCSMA/CA	Effect of ILA-CSMA
SF7–SF8	Frequent re-entry into contention can dominate short-term access	Hybrid sensing reduces hidden collisions while ToA-aware backoff limits excessive contention advantage
SF9–SF10	Medium-airtime packets suffer from both low-SF contention and high-SF collision exposure	Combined sensing and backoff stabilize success probability across the middle SFs
SF11–SF12	Long airtime increases collision cost and retransmission penalty	Larger ToA-weighted windows reduce repeated overlap and improve high-SF delivery opportunity

**Table 4:** Representative performance summary under heavy congestion.

Metric	Standard CSMA/CA	ILA-CSMA
Packet delivery ratio	Baseline	About +20 percentage points
Jain fairness index	Lower under high load	Above the 0.85 reference line
Energy per successful packet	Baseline	22% of baseline
Conditional packet delay at 2000 nodes	18.5 s	8.2 s

## Data Availability

Data are contained within the article.

## Abbreviations

The following abbreviations are used in this manuscript:

ADR	Adaptive Data Rate
CAD	Channel Activity Detection
CSMA/CA	Carrier Sense Multiple Access with Collision Avoidance
ILA-CSMA	Interference-Limit-Aware CSMA
IoT	Internet of Things
JFI	Jain Fairness Index
LoRa	Long Range
LoRaWAN	Long Range Wide Area Network
LR-FHSS	Long-Range Frequency-Hopping Spread Spectrum
MAC	Medium Access Control
PDR	Packet Delivery Ratio
PHY	Physical Layer
RSSI	Received Signal Strength Indicator
SF	Spreading Factor
ToA	Time on Air
